# Extracellular Vesicle Characteristics in β-thalassemia as Potential Biomarkers for Spleen Functional Status and Ineffective Erythropoiesis

**DOI:** 10.3389/fphys.2018.01214

**Published:** 2018-08-30

**Authors:** Carina Levin, Ariel Koren, Annie Rebibo-Sabbah, Naama Koifman, Benjamin Brenner, Anat Aharon

**Affiliations:** ^1^Pediatric Hematology Unit, Emek Medical Center, Afula, Israel; ^2^The Ruth and Bruce Rappaport Faculty of Medicine, Technion – Israel Institute of Technology, Haifa, Israel; ^3^Department of Hematology and Bone Marrow Transplantation, Rambam Health Care Campus, Haifa, Israel; ^4^Department of Chemical Engineering and The Russell Berrie Nanotechnology Institute, Technion – Israel Institute of Technology, Haifa, Israel

**Keywords:** thalassemia, extracellular vesicle, microparticle, hypersplenism, HSP70

## Abstract

β-thalassemia major (β-TM) is a therapeutically challenging chronic disease in which ineffective erythropoiesis is a main pathophysiological factor. Extracellular vesicles (EVs) are membrane-enclosed vesicles released by cells into biological fluids; they are involved in intercellular communication and in multiple physiological and pathological processes. The chaperone heat-shock protein 70 (HSP70), which is released from cells via EVs, aggravates ineffective erythropoiesis in β-TM. We propose that β-TM EVs may show specific signatures, reflecting disease mechanisms, stages and severity. Our study aims were to define EV profiles in β-TM patients, investigate the influence of hypersplenism and splenectomy on EV features, and explore the association of circulating EVs with ineffective erythropoiesis and iron-overload parameters. We characterized circulating EVs in 35 transfusion-dependent β-thalassemia patients and 35 controls using several techniques. Nanoparticle-tracking analysis revealed increased EV concentration in patients vs. controls (*P* = 0.0036), with smaller EV counts and sizes in patients with hypersplenism. Flow cytometry analysis showed lower levels of RBC and monocyte EVs in patients vs. controls. RBC-EV levels correlated with patient hematocrit, reflecting degree of anemia. The procoagulant potential of the EVs evaluated by flow cytometry revealed lower levels of endothelial protein C receptor-labeled EVs in patients vs. controls, and increased tissue factor-to-tissue factor pathway inhibitor-labeled EV ratio in splenectomized patients, suggesting a hypercoagulable state. Protein content, evaluated in EV pellets, showed increased levels of HSP70 in patients (*P* = 0.0018), inversely correlated with transfusion requirement and hemoglobin levels, and positively correlated with reticulocyte, erythropoietin and lactate dehydrogenase levels. This first description of EVs in patients with hypersplenism reveals the spleen’s importance in EV physiology and clearance. Circulating EV-HSP70 levels were associated with markers of ineffective erythropoiesis, hemolysis and hematological disease severity. EV analysis in β-TM—reflecting spleen status, hypercoagulability state and ineffective erythropoiesis—may serve as a biomarker of disease dynamics, supporting both anticipation of the risk of complications and optimizing treatment.

## Introduction

β-thalassemia syndromes are a group of hereditary disorders characterized by a genetic deficiency in the synthesis of β-globin chains. The imbalance in α- to β-globin chains leads to ineffective erythropoiesis, defined as the inability to produce an adequate number of red blood cells (RBC) in the presence of increased immature erythroid precursors in the bone marrow (Rivella, 2009), and severe anemia. Patients with β-thalassemia major (β-TM) require blood transfusions for life and may develop severe dysfunctions in major organs and systems. Transfusional iron load and enhanced iron absorption result in iron accumulation and deposition in vital organs with harmful effects. As there is no physiological mechanism to excrete iron from the body, patients require lifelong chelation therapy to prevent iron overload (Rund and Rachmilewitz, 2005). Reduction of erythroid cell lifespan, free-iron toxicity, and deposition of excess iron in vital organs are the major factors responsible for the functional and physiological abnormalities found in patients with thalassemia (Rund and Rachmilewitz, 2005; Koren et al., 2010; Breuer et al., 2012; Rund, 2016).

From a therapeutic viewpoint, two groups of thalassemic patients can be defined: those with non-transfusion-dependent thalassemia (NTDT) and those with transfusion-dependent thalassemia (TDT), principally β-TM patients.

Hypersplenism, defined as enlargement of the spleen causing premature destruction of blood cells, capable of aggravating or inducing anemia, thrombocytopenia, leukopenia or a combination of these (Brousse et al., 2014), is a common feature in thalassemic patients. Splenectomy can ameliorate pancytopenia and reduce blood-transfusion requirements. Although splenectomy is commonly performed in patients with thalassemia, it is associated with increased risk of bacterial infections, thrombotic complications and pulmonary hypertension (Du et al., 1997; Eldor and Rachmilewitz, 2002; Cappellini et al., 2012; Musallam et al., 2012; Sakran et al., 2012).

Extracellular vesicles (EVs) are membrane-enclosed vesicles secreted by cells into biological fluids via membrane “shedding” and secretion (Toth et al., 2007). EVs can stem from nearly every type of cell and contain bioactive molecules, such as proteins, phospholipids and nucleic acids, which characterize the cell from which they originate (Cocucci et al., 2009; Kalra et al., 2016). There are three main types of EVs: exosomes (diameter range 30–100 nm) formed in the multivesicular bodies, microvesicles/microparticles (MPs) (0.1–l μm) which are cell-surface plasma-membrane-derived particles, and apoptotic bodies >1 μm in size). These EVs differ not only in size but also in their release mechanisms (Abels and Breakefield, 2016).

Extracellular vesicles have specific characteristics and pathophysiological roles, and can be used for outcome prediction in several diseases, including sickle cell anemia (Shet et al., 2003; Westerman et al., 2008; van Beers et al., 2009; Camus et al., 2015). Previous studies in patients with β-thalassemia have demonstrated increased levels of MPs in both NTDT (Pattanapanyasat et al., 2007; Westerman et al., 2008; Chaichompoo et al., 2012) and in TDT; in addition with a higher number of MPs originated from RBCs and platelets (Tantawy et al., 2013; Elsayh et al., 2014; Agouti et al., 2015). After splenectomy, blood cell counts and circulating MPs increase. This has been observed in patients with immune thrombocytopenia (Fontana et al., 2008; Sewify et al., 2013), β-thalassemia intermedia (β-TI) patients (Pattanapanyasat et al., 2007; Habib et al., 2008; Westerman et al., 2008; Klaihmon et al., 2017) and TDT patients; in the latter group, splenectomy has been associated with higher levels of MPs (Tantawy et al., 2013; Elsayh et al., 2014; Agouti et al., 2015) and increased procoagulant activity of platelet MPs (Agouti et al., 2015). However, there have been no studies characterizing EVs in thalassemic patients with hypersplenism.

Despite extensive knowledge of the molecular defects causing β-thalassemia, the mechanisms responsible for ineffective erythropoiesis are not fully understood (Arlet et al., 2014). In addition to apoptosis of erythroid precursors, limited cell differentiation that decreases RBC production has been proposed; abnormal exposure of phosphatidylserine (PS) is considered a principal feature of apoptotic RBC precursors, but it can also be observed in β-thalassemia mature erythrocytes (Libani et al., 2008).

Recent studies have suggested that during normal erythropoiesis, the chaperone heat-shock protein 70 (HSP70), which is involved in cellular protein homeostasis and plays a key role in erythropoiesis, translocates into the nucleus and protects the erythroid transcription factor GATA1 from caspase 3 cleavage and degradation (Ribeil et al., 2007). In β-thalassemia erythroblasts, the free α-globin chains interact with HSP70, sequestering it in the cytoplasm and leaving GATA1 unprotected. Its consequent degradation results in end-stage maturation arrest and apoptosis, which further aggravates the ineffective erythropoiesis observed in β-TM (Arlet et al., 2014; Sankaran and Weiss, 2015; Makis et al., 2016). In addition, HSP70 can be released by stressed cells as free soluble protein or via EVs; extracellular membrane-bound HSP70 is involved in immune system stress response (De Maio and Vazquez, 2013), and tumor-related EV HSP70 also induces muscle catabolism and wasting in a mouse model (Zhang et al., 2017). MPs released from β-TI RBC have been found to contain specific proteins, including HSP70 (Ferru et al., 2014). However, the EV content of HSP70 and its association with clinical and laboratory manifestations of TDT patients have never been studied.

Here we used several methods to characterize the circulating EVs in TDT patients, investigate the influence of hypersplenism and splenectomy on EV features, and explore the association of circulating EV HSP70 levels with ineffective erythropoiesis and iron overload.

## Materials and Methods

The study was conducted between the years 2013 and 2017. TDT patients treated at the Pediatric Hematology Unit of Emek Medical Center were compared with healthy controls. The study was approved by the Ethics Committee, Emek Medical Center, Afula, Israel (EMC: 0142-12) and conducted in accordance with Good Clinical Practice guidelines and the Declaration of Helsinki. Written informed consent was obtained from all adult research participants and from the parents/legal guardians of all non-adult participants. Patients under 3 years of age, with chronic active hepatitis C or with HIV, and pregnant women were excluded from the study. Patients were treated under a standard protocol of regular blood transfusions every 2–3 weeks and chelation therapy, based on Israeli and Thalassemia International Federation guidelines; no central lines were used in our patients.

Complete blood count, including white blood cells (WBC), hemoglobin (Hb), hematocrit (HCT), platelets, reticulocyte percentage and reticulocyte Hb content, iron, transferrin and transferrin saturation, lactate dehydrogenase, erythropoietin and ferritin, performed on the same day or at the closest possible date to EV sampling (up to 2 months for ferritin), were studied and used for correlations with the EV study results.

Genetic mutations, demographic data, clinical manifestation, thrombotic events and laboratory data—including mean last-5-year Hb levels and annual transfusion requirement (mL packed cell/kg weight per year), were obtained from patients’ files.

### EV Isolation

The blood samples were collected after overnight fasting. Patients’ blood samples were obtained on the day of, and before routine blood transfusion through an intravenous 20–22 gauge cannula; after tourniquet release, the first tubes were used for routine samples. In controls, the samples were obtained through 20–22 gauge cannula or butterfly needles after discarding the first few milliliters of blood. Blood was collected in sodium citrate (1:10) for nanoparticle-tracking analysis (NTA) and flow cytometry studies, and in EDTA tubes for the HSP70 ELISA. Blood samples were centrifuged twice for 15 min at 1,500*g*. The platelet-poor plasma (PPP) was frozen in aliquots (−80°C). EV pellets were isolated from thawed PPP by centrifugation (1 h, 20,000 *g* at 4°C, rediluted in PBS and recentrifuged) (Tzoran et al., 2015).

### EV Characterization

Extracellular vesicles count, cellular origin and membrane antigens were evaluated in PPP by NTA and flow cytometry.

Nanoparticle-tracking analysis is a method for the assessment of particle size (in the range of 50–2000 nm) and concentration in liquids, that relates the rate of Brownian motion to particle size (Gardiner et al., 2013). NTA was performed in scattering and fluorescent mode using a NanoSight^®^ NS500-Zeta HSB system with a CMOS camera and 638-nm laser (Malvern Instruments); Alexa Fluor 647 annexin-V (BioLegend, San Diego, CA, United States) was used for fluorescent analysis.

Flow cytometry was performed using a previously described protocol (Tzoran et al., 2015) with a flow cytometer CyAn ADP analyzer (Beckman Coulter). Briefly, forward and side scatter were set on logarithmic scales, the gate for EV analysis was set at < 1 μm using Megamix beads (0.5, 0.9, 3 μm, Biocytex, Marseille, France) and 0.78-μm beads (BD Biosciences). EV concentrations were calculated using 7.5-μm count beads. To determine the presence of PS, fluorescein isothiocyanate (FITC)-labeled annexin V (Bender MedSystems) was used. To evaluate EV cellular origin, they were labeled with conjugated mouse anti-human: phycoerythrin (PE)-CD41 (platelet, Biolegend, San Diego, CA, United States), FITC-CD14 (monocytes, IQ Products, Netherlands), PE-CD11a (leukocytes), PE-CD62p (activated platelets), FITC-CD31 (endothelial cells), PE-CD235 (RBC marker glycophorin A^+^) and FITC and PE-IgG1κ isotype controls (BD Biosciences, San Jose, CA, United States). The results are expressed in EV/μL, and percentage of labeled EVs after subtracting isotype-matched positive control events.

Extracellular vesicles morphology was imaged by cryogenic temperature–transmission electron microscopy (cryo-TEM): EV PPP and pellets from a subset of randomly selected controls and patients were characterized by cryo-TEM. Specimens were prepared as described previously (Issman et al., 2013), transferred into a Gatan 626DH cryo-holder and equilibrated below -180°C. Micrographs were recorded by an FEI Ceta 16M, a 4k × 4k pixel, high-resolution CCD camera on a Talos 200C (FEI) transmission electron microscope operated at 200 kV.

### EV Content of Apoptosis-Related Proteins

Extracellular vesicles pellets were obtained from 2 mL of a pool of 4 individuals (0.5 mL PPP each) in each study group. The expression level of 43 apoptosis-related proteins was screened by apoptotic protein array (RayBiotech) performed according to the manufacturer’s instructions as previously described (Shomer et al., 2013). Slides were then completely dried, and scanned at 5-μm resolution on the Agilent G2565BA Microarray Scanner (Agilent Technologies, Santa Clara, CA, United States) and analyzed using TotalLab software. Results were normalized to healthy controls. For HSP70 content, EV pellets from 0.5 mL PPP, obtained from blood collected in EDTA tubes and after solubilization of EVs using lysis buffer (RayBiotech) from each individual, were evaluated in duplicates by ELISA (ELH-HSP70, RayBiotech) according to the manufacturer’s instructions.

### EV Antigens Involved in Coagulation

To determine the procoagulant potential of the EVs, each sample was labeled with fluorescent antibodies against tissue factor (TF) and tissue factor pathway inhibitor (TFPI) (American Diagnostics, Los Angeles, CA, United States), thrombomodulin (BD Biosciences Pharmingen, San Diego, CA, United States) and endothelial protein C receptor (EPCR) (Santa Cruz Biotechnology, Dallas, TX, United States). PPP (50 μL) was used for labeling with one or more specific antibodies; after incubation for 30 min at room temperature in the dark suspended in 300 μL phosphate-buffered saline (PBS) containing 0.02% formaldehyde, the labeled plasma was scanned by CyAn ADP analyzer (Aharon et al., 2009).

### Statistical Analysis

Data were analyzed using GraphPad-5 software. Continuous variables were reported as mean ± SD and as median and interquartile range. Differences between controls and patients were tested using Mann–Whitney test; to compare subgroups, Kruskal–Wallis test was performed with subsequent Dunn’s multiple comparison test. To further reduce the risk of false discovery due to multiple testing, the Benjamini–Hochberg false discovery rate method was used to adjust the *P*-values. Spearman’s correlation test was used to correlate EV results with the patients’ laboratory parameters. For all analyses, two-tailed test with significance *P* < 0.05 was used.

## Results

### Study Population

Blood samples were collected from 35 healthy controls and 35 TDT patients. There was no significant difference in age or gender between controls and patients or among patient subgroups. The characteristics of the controls were: mean age 21 ± 9 years (range 7–38), 17 males and 18 females, and for the patients: mean age 22 ± 7.3 years (3–39), 18 males and 17 females. All TDT patients were genetically homozygous or compound heterozygous for the following mutations: HBB:c.93-21G > A; HBB:c.114G > A; HBB:c.118C > T; HBB:c.316-106C > G; HBB:c.78A > C; HBB:c.92+6T > C; HBB:c.92+5G > C; HBB:c.25_26delAA; HBB:c.92+1G > A. Three patient subgroups were considered based on spleen status: (i) hypersplenism (Hy); (ii) no hypersplenism (No-Hy); (iii) splenectomized (Sp). Hypersplenism was defined as patients with cytopenia requiring ≥ 200 mL packed RBC per kg body weight per year. Five patients with hypersplenism required a splenectomy during the study period, after which EVs were reevaluated; consequently, patient subgroups included a total of 40 investigations. Subgroup characteristics were Hy: *n* = 9, age 23.8 ± 5 years (range 13–29); No-Hy: *n* = 18, age 19.8 ± 8 years (3–33); Sp: *n* = 13, age 26.7 ± 5 years (19–39).

**Table 1:** Patients’ blood laboratory values.

Parameter and units	Normal value	β-thalassemia patient subgroups				*P*-value between sub-groups	Adjusted *P*-value
		Hy *N* = 9	No-Hy *n* = 18	Sp *n* = 13	Total *n* = 40		
WBC K/μL	4.5–11.5	3.6 ± 1	6.9 ± 1.9	16 ± 9	9.6 ± 7.3	<0.0001 ‡	0.0002
Hb g/dl	12–15	7 ± 1	8.4 ± 0.7	7.8 ± 0.7	7.9 ± 0.7	0.0034 ‡‡	0.007
HCT %	37–47	20.1 ± 3.2	24 ± 1.9	23.6 ± .2.1	23.2 ± 2.8	0.0035 ‡;‡‡	0.007
PLT K/μL	150–450	172 ± 27	295 ± 92	777 ± 387	450 ± 344	<0.0001 ‡	0.0002
Ferritin ng/mL	22–322	2549 ± 2000	1945 ± 1190	2157 ± 1330	2079 ± 1389	0.9038	0.9038
Serum iron μg/dL	40–145	215 ± 70	214 ± 62	194 ± 23	211 ± 59	0.6875	0.7639
Transferrin mg/dL	200–360	125 ± 16	145 ± 28	141 ± 24	139 ± 25	0.3148	0.3935
Erythropoietin mIU/mL	4–29	381 ± 114	134 ± 85	91.1 ± 56	164 ± 134	0.0008 ‡; ‡‡	0.0027
Ret %	0.5–2.5	1.78 ± 1.5	1.18 ± 0.9	4.99 ± 3.3	2.7 ± 2	0.0049 ¥	0.0082
LDH U/L	230–480	860 ± 536	367 ± 90	361 ± 106	457 ± 304	0.0407 ‡	0.0581

*Data are expressed as mean ± SD. WBC, white blood cells; Hb, hemoglobin; HCT, hematocrit; PLT, platelets; Ret, reticulocytes; LDH, lactate dehydrogenase. Adjusted P-values for multiple testing were calculated using the Benjamini–Hochberg false discovery rate method. Significant P-values between patient subgroups: ‡ between the three patient subgroups; ‡ between patient subgroups Hy and Sp; ‡‡ between patient subgroups Hy and No-Hy; ¥ between patient subgroups Sp and No-Hy; n, number of controls or patients studied per parameter.*

### WBC, Platelet Counts and Hb Levels Decrease While Erythropoietin and Lactate Dehydrogenase Levels Increase in Hy Patients

**FIGURE 1:**
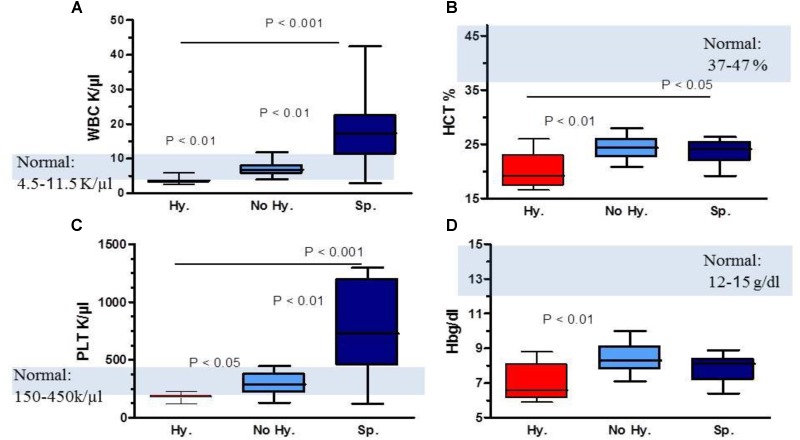
Comparison of complete blood count parameters in patient subgroups. **(A)** WBC levels, **(B)** hematocrit (HCT) levels, **(C)** platelet (PLT) count and **(D)** hemoglobin (Hb) levels. Data are displayed as median (horizontal bar), range from 25th to 75th percentile (box), and extremes of distribution from 10th and 90th percentiles (error bar). Gray area represents normal range values. To compare different subgroups, Kruskal–Wallis test was performed with subsequent Dunn’s multiple comparison test. For all analyses, two-tailed test with significance *P* < 0.05 was used.

Laboratory characteristics of patients and controls are summarized in **Table 1**. Significant statistical differences for WBC, Hb and platelet levels were found among patient subgroups (**Figure 1** and **Table 1**). In the Hy group, WBC and platelet counts were below normal values (**Figures 1A,C**). While, in Sp patients WBC and platelet counts were higher than normal. In all three patient subgroups, the mean pre-transfusion Hb and HCT were below normal values (**Figures 1B,D**).

In all three patient subgroups, iron and ferritin levels were higher than normal values and transferrin was below normal values; no significant statistical differences were found among patient subgroups. In the Hy subgroup, erythropoietin and lactate dehydrogenase levels were significantly elevated while reticulocyte count was higher in the Sp subgroup (**Table 1**).

### Morphological Characterization of EVs by Cryo-TEM

**FIGURE 2:**
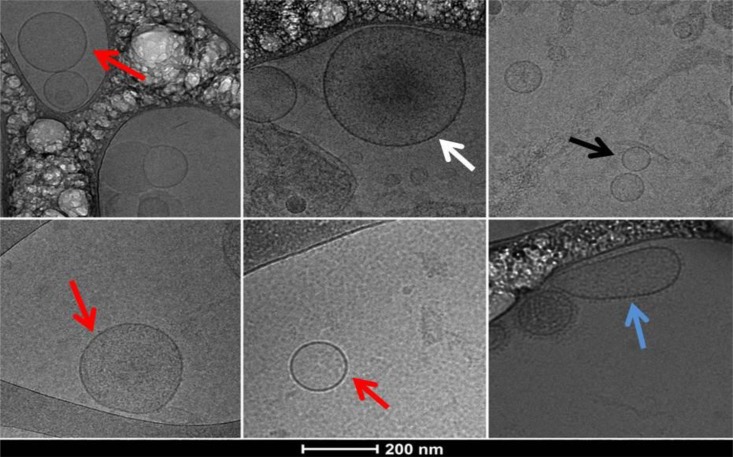
Cryo-TEM images of extracellular vesicles (EVs) obtained from PPP pellet sample of a patient with hypersplenism showing a heterogeneous population of EVs. Typical spherical EVs: white arrow a large EV (microparticle) with dense intravesicular content; red arrows middle size EVs; black arrow small exosomes (<100 nm); blue arrow middle size EV with elongated shape and visible external membrane elements.

In all samples studied—EVs from PPP and from pellets—from the different study groups, we observed a heterogeneous population of EVs, with a wide size range; large MPs, with dense intravesicular content, middle size EVs (100–300 nm) and small exosomes (<100 nm). Most particles were spherical but varied in membrane appearance: some had a granular morphology while others had a smooth membrane. In addition, some of the vesicles showed an oblate rather than spherical shape (**Figure 2**). Cryo-TEM micrographs confirmed that our measurements by NTA and flow cytometry, and pellets used for protein analysis, consisted of vesicles with a closed membrane carrying intravesicular content and were not cell debris.

### EV Concentration, Size and Origin Are Associated to Spleen Status in TDT Patients

Nanoparticle-tracking analysis was used to assess concentration and size distribution of particles in the diameter range of 50–1,000 nm, and flow cytometry to assess particles of ≥300 nm diameter.

**FIGURE 3:**
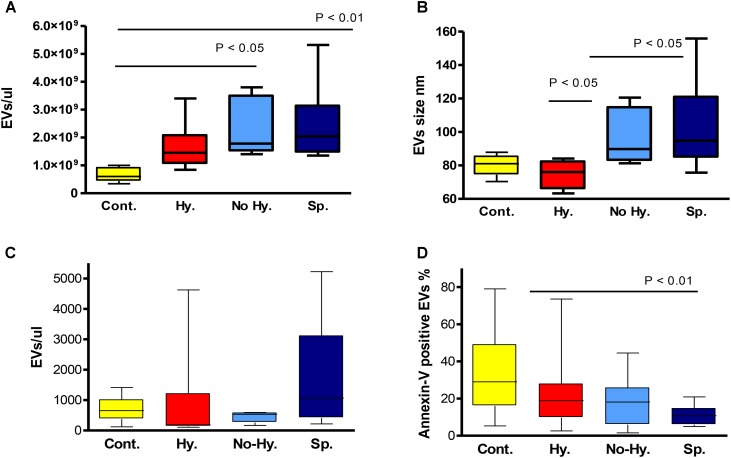
Nanoparticle-tracking analysis-determined EV count **(A)** and size **(B)**. Controls *n* = 5, hypersplenism (Hy) *n* = 7, No-Hy *n* = 6, splenectomized (Sp) *n* = 9. Flow-cytometry-determined EV count (EV/μL) **(C)** and percentage of annexin-V-positive EVs **(D)**. Controls *n* = 17–20, Hy *n* = 7–9, No-Hy *n* = 8–11, Sp *n* = 11–13. Data are displayed as median (horizontal bar), range from 25th to 75th percentile (box), and extremes of distribution from 10th to 90th percentiles (error bar). To compare different subgroups, Kruskal–Wallis test was performed with subsequent Dunn’s multiple comparison test. For all analyses, two-tailed test with significance *P* < 0.05 was used.

Nanoparticle-tracking analysis was performed for a subset of randomly selected patient and control samples and demonstrated a strong increase in EV concentration in the former (**Table 2**). Significant differences were observed in the comparison of patient subgroups. The lowest EV count and smallest size were observed in the Hy group and the highest count and size in the Sp group (**Table 2** and **Figures 3A,B**). The number of annexin-V-labeled EVs measured by NTA was similar in patients and controls, but higher in the Hy vs. No-Hy group (**Table 2**).

The count of large EVs, measured by flow cytometry, was slightly higher in the Sp subgroup (*P* = 0.051) (**Table 2** and **Figure 3C**). The percentage of annexin-V-labeled EVs measured by flow cytometry was significantly lower in patients than in controls (*P* = 0.0036), with no significant differences among patient subgroups (**Table 2** and **Figure 3D**). However, the lowest levels of annexin-V-labeled large EVs were observed in the Sp group and highest levels in the Hy patients (**Table 2**).

**FIGURE 4:**
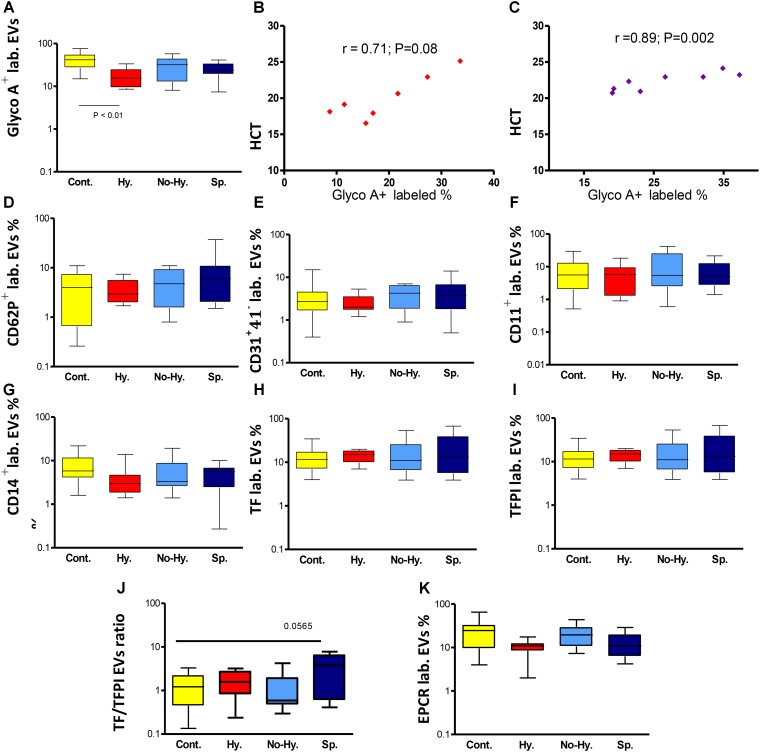
Flow cytometry of EVs. **(A)** Percentage of RBC-derived (glycophorin A^+^-labeled [GlycoA^+^ labeled.]) EVs in the different groups. Correlation between percentage of RBC-derived EVs and HCT in the Hy **(B)** and Sp **(C)** groups (*n* = 7–8). Flow cytometry of activated platelet-derived EVs (CD62P ^+^ lab.) **(D)**, endothelial cell-derived EVs (CD31^+^41^−^ lab.) **(E)**, leukocyte-derived EVs (CD11a^+^ lab.) **(F)** and monocyte-derived EVs (CD14^+^ lab.) **(G)**. Flow cytometry for procoagulant and anticoagulant potential of EVs. **(H)** Tissue factor (TF)-labeled EVs. **(I)** TF pathway inhibitor (TFPI)-labeled EVs. **(J)** TF/TFPI ratio. **(K)** Endothelial protein C receptor (EPCR)-labeled EVs. Controls *n* = 17–20, Hy *n* = 7–9, No-Hy *n* = 8–11, Sp *n* = 11–13. Data are displayed as median (horizontal bar), range from 25th to 75th percentile (box), and extremes of distribution from 10th to 90th percentiles (error bar). To compare subgroups, Kruskal–Wallis test was performed with subsequent Dunn’s multiple comparison test. Spearman’s correlation test was used to correlate EV results with the patients’ laboratory parameters. For all analyses, two-tailed test with significance *P* < 0.05 was used.

Extracellular vesicles cellular origins, analyzed by flow cytometry, are shown in **Table 2**. The percentage of RBC-derived EVs was significantly lower in patients than in controls without significant difference among patient subgroups (**Table 2**). However, the lowest levels were observed in Hy patients (**Figure 4A**). The percentage of RBC-derived EVs was correlated with HCT levels in the Hy and Sp subgroups (**Figures 4B,C**). Despite receiving periodic blood transfusions, patients had pronounced anemia and a lower percentage of RBC-derived EVs than healthy controls. The Hy group showed the lowest Hb level as well as the lowest level of RBC-derived EVs.

The percentage of platelet-derived EVs (CD41^+^) and the percentage of activated platelet-derived EVs (CD62P^+^) did not differ significantly between patients and controls or among patient subgroups (**Table 2**). However, in patient subgroups, the lowest levels of CD62P^+^ were observed in the Hy group and the highest in the Sp group (**Table 2** and **Figure 4D**). The percentage of endothelial cell-derived EVs (CD31^+^CD41^−^) and leukocyte-derived EVs (CD11a^+^) did not differ significantly between patients and controls. However in patient subgroups, the lowest levels were observed in Hy patients and the highest in the Sp group (**Table 2** and **Figures 4E,F**). The percentage of monocyte-derived EVs (CD14^+^) was lower, albeit not significantly so, in patients than in controls, and Hy patients showed the lowest levels (**Table 2** and **Figure 4G**).

### EV Procoagulant and Anticoagulant Potential

The percentage of TF- and TFPI-labeled EVs did not differ significantly between patients and controls (**Table 2** and **Figure 4H**). However, the highest percentage of TF-labeled and lowest percentage of TFPI-labeled EVs were observed in Sp patients, and the opposite was seen in Hy patients (**Table 2** and **Figure 4I**).

The TF/TFPI ratio was particularly high in Sp patients (**Table 2** and **Figure 4J**). The percentage of EPCR-labeled EVs was significantly lower in the total patient group than in controls (**Table 2**), with lowest levels found in the Hy subgroup. In the No-Hy subgroup, the percentage of EPCR-labeled EVs was similar to that in normal controls (**Figure 4K**).

### EV Apoptotic Protein Content

**FIGURE 5:**
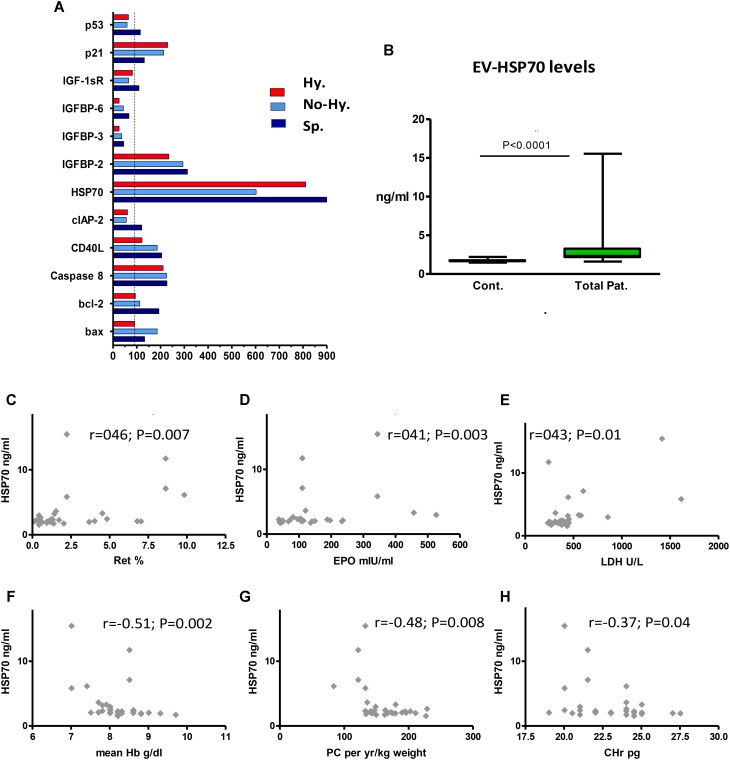
Extracellular vesicles content of apoptosis-related proteins and correlation between EV HSP70 and blood parameters. **(A)** Human apoptotic protein array results. The protein content of pooled EV specimens (pellets from 4 individual samples per group) was studied. Protein levels are expressed as percentage of control values. IGF factor 1 receptor (IGF-1sR), IGF binding protein (IGFBP), baculoviral inhibitor of apoptosis repeat-containing protein 2 (cIAP-2), CD40 ligand (CD40L). **(B)** HSP70 levels in control and patient EVs. EV pellets obtained from each patient (*n* = 32) were evaluated in duplicate by ELISA. Data are displayed as median (horizontal bar), range from 25th to 75th percentile (box), and extremes of distribution from 10th to 90th percentiles (error bar). Differences between controls and total patients were subjected to Mann–Whitney test. **(C–F)** Correlation between EV-HSP70 levels and blood parameters (*n* = 30–32). Reticulocyte (Ret) percentage **(C)**, erythropoietin (EPO) level **(D)**, and lactate dehydrogenase (LDH) level **(E)**, mean last-5-year Hb levels **(F)**, annual transfusion requirement (mL packed cell [PC]/kg weight per year) **(G)**, and reticulocyte hemoglobin content (CHr) **(H)**. Spearman’s correlation test was used to correlate HSP70 results with the patients’ laboratory parameters. For all analyses, two-tailed test with significance *P* < 0.05 was used.

Several differences in the apoptotic protein profiles were revealed between patients and controls; nevertheless, a uniform pattern of protein levels was observed in all patient groups. Increased levels of HSP70, IGF binding protein 2 (IGFBP-2) and caspase 8, and decreased levels of IGFBP-3 and IGFBP-6 were found (**Figure 5A**).

### HSP70 Is Increased in Circulating EVs From TDT Patients and Is Associated With Markers of Ineffective Erythropoiesis

The increased HSP70 levels screened by the protein array were evaluated in the entire study cohort by ELISA. We found significantly higher levels of HSP70 in patient vs. healthy control EVs (**Table 2** and **Figure 5B**), with a non-significant difference among patient subgroups. However, the highest levels were found in Hy patients (**Table 2**).

**Table 2:** Extracellular vesicles (EV) characteristics including EV count, size and cellular origin and membrane antigens analyzed by NTA and flow cytometry, and HSP70 levels in controls, total patients and patient subgroups.

Parameter and units	Cont.	β-thalassemia patient subgroups				*P*-value Cont. vs. Total	Adjusted *P*-value Cont. vs. Total	*P*-value sub-groups	Adjusted *P*-value sub- groups
		Hy	No-Hy	Sp	Total				
**NTA**	***n* = 5**	***n* = 7**	***n* = 6**	***n* = 9**	***n* = 22**				
EV/μL E+09	0.66 ± 0.2	1.7 ± 0.8	2.26 ± 0.9	2.4 ± 1	2.15 ± 1.1	0.0008	0.0036	0.3328	0.5971
Mean size nm	80.16 ± 6.4	74.39 ± 7	95.7 ± 15	103 ± 25	91.9 ± 22	0.2236	0.575	0.0053 ‡;‡‡	0.0238
Annexin + EV/μL E+05	2.08 ± 0.6	17 ± 15	0.97 ± 3	4.08 ± 5	7.5 ± 11	0.9601	0.962	0.0166	0.0501
Mean size (nm) EVs annexin +	295.6 ± 140	279 ± 79	237 ± 47	265 ± 95	261 ± 74	0.9623	0.962	0.9396	0.9396
**Flow cytometry**	***n* = 17–20**	***n* = 7–9**	***n* = 8–11**	***n* = 11–13**	***n* = 26–33**				
EV/μL	702.7 ± 392	886.5 ± 96	433 ± 167	1831 ± 1602	1151 ± 1426	0.7302	0.962	0.0167 #; ‡	0.0501
Annexin^+^ EV %	33.67 ± 19	23.6 ± 22	18 ± 13	11.38 ± 5	16.8 ± 14	0.0007	0.0036	0.0025	0.015
Glyco A^+^ EV %	41.9 ± 16	17 ± 8	29.5 ± 16	25 ± 9	24.3 ± 12	0.0004	0.0036	0.0013 #	0.0117
CD41^+^ EV %	40.9 ± 22	37 ± 24	36.4 ± 9	43.6 ± 22	39 ± 20	0.8468	0.962	0.9206	0.9396
CD62P^+^ EV %	4 ± 3	3.6 ± 2	5.2 ± 3	9 ± 10	6.1 ± 7	0.3355	0.624	0.5311	0.7354
CD31^+^41^−^ EV%	4 ± 4	2.5 ± 1.4	4.1 ± 2.3	5 ± 4	4.1 ± 3	0.6891	0.962	0.5183	0.7354
CD14^+^ EV %	7.4 ± 5	4 ± 3	5.8 ± 5	4.8 ± 2.9	4.9 ± 3	0.0414	0.149	0.0311 #	0.08
CD11^+^ EV %	9.1 ± 9	6.4 ± 5	12 ± 14	7.3 ± 6	8.7 ± 9	0.9447	0.962	0.9045	0.9396
TF^+^ EV %	13.5 ± 10	14.1 ± 4	17 ± 15	22 ± 20	18.2 ± 16	0.6146	0.962	0.8106	0.9396
TFPI^+^ EV %	12 ± 8	14.6 ± 10	12.7 ± 8	9.8 ± 9	12.1 ± 9	0.7848	0.962	0.3649	0.5971
TF/TFPI ratio	1.3 ± 1.4	1.7 ± 1	1.2 ± 1.3	3.8 ± 2	2.3 ± 2	0.2005	0.575	0.0565	0.1271
EPCR^+^ EV %	25.2 ± 17	10 ± 4	20 ± 11	12.8 ± 7	14.5 ± 9	0.0316	0.1156	0.031	0.0548
TM^+^ EV %	8.8 ± 11	11.9 ± 11	7.4 ± 5	13.5 ± 12	11.2 ± 10	0.3467	0.624	0.6416	0.8249
**EV HSP70 ELISA**	***n* = 11**	***n* = 6**	***n* = 14**	***n* = 12**	***n* = 32**				
HSP70 (ng/mL)	1.7 ± 0.2 [1.7; 1.5-1.7]	5.27 ± 5 [3.2; 2-3.2]	2.2 ± 0.3 [2; 2-2.4]	3.9 ± 2 [2.4; 2.1-4.9]	3.46 ± 3 [2.33; 2-3.2]	0.0001	0.0018	0.031	0.08

*Data are expressed as mean ± SD, EV HSP70 values are expressed in SD and median and interquartile range []. NTA studies are expressed in EV/μL, and mean particle size in nm. Flow cytometry studies are expressed in EV/μL and percent of positive labeled EVs. EV-HSP70 levels in EV pellets obtained from 0.5 mL PPP were evaluated in duplicate by ELISA. Adjusted P-values for multiple testing were calculated using the Benjamini–Hochberg false discovery rate method. Significant P-values between subgroups: # between controls and Hy patients; ‡ between patient subgroups Hy and Sp; ‡‡ between patient subgroups Hy and No-Hy; n, number of controls or patients studied per parameter.*

HSP70 showed a moderate positive correlation with patients’ reticulocyte count, erythropoietin levels and lactate dehydrogenase (**Figures 5C–E**), and a negative correlation with Hb levels obtained on the same day as the EV sampling, mean last-5-year Hb levels, annual transfusion requirement (mL/kg weight) and reticulocyte Hb content (**Figures 5F–H**). However, no correlation was found with iron-overload parameters (data not shown).

**FIGURE 6:**
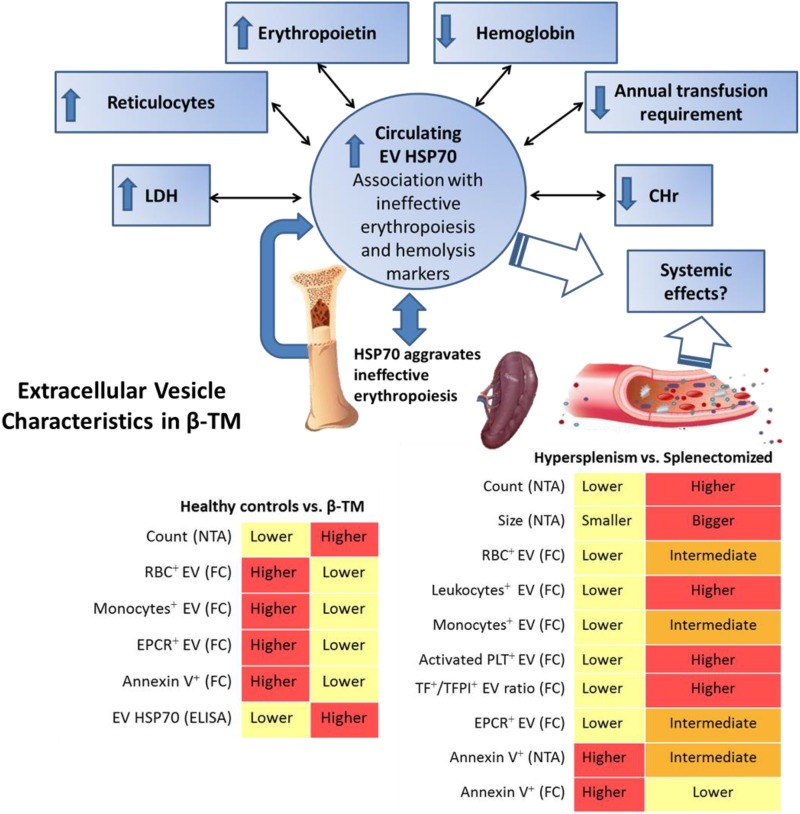
Summarizing diagram of EV characteristics found in transfusion-dependent β-thalassemia patients. Nanoparticle-tracking analysis (NTA) revealed highly increased EV concentration in the whole cohort of patients compared to healthy controls. Flow cytometry (FC) revealed lower levels of RBC^+^-, monocyte^+^-, annexin^+^ and endothelial protein C receptor (EPCR)^+^ EVs in patients compared to controls. Circulating EV-HSP70 levels measured by ELISA were highly increased in our patients. EV-HSP70 levels were inversely correlated with blood-transfusion requirement, mean hemoglobin (Hb) levels and reticulocyte Hb content (CHr), and positively correlated with reticulocyte, erythropoietin and lactate dehydrogenase levels reflecting disease hematological severity and ineffective erythropoiesis. In patients with hypersplenism compared to those with no-hypersplenism and splenectomized patients, decreased EV concentration and smaller size were found by NTA, and lower RBC^+^-, leukocyte^+^-, monocyte^+^-, activated platelet^+^- (PLT), TF^+^/TFPI^+^-, and EPCR^+^-EVs were evaluated by FC. Overall, specific EV signatures were detected in thalassemic patients reflecting different disease stages, such as those related to spleen functional status, hypercoagulability state and ineffective erythropoiesis.

## Discussion

The current study demonstrates specific EV patterns in TDT patients, and the relationship between EV features and patients’ clinical and laboratory parameters. There was a sharp increase in EV concentration in patients compared to healthy individuals (**Figure 6**), while the lowest EV count and size were observed in patients with hypersplenism. We show that spleen status affects patient’s blood cell count as well as EV patterns. The role of the spleen in modulating EVs under physiological and pathological conditions warrants further study.

The large EVs in patients differed from those in controls in that a lower percentage of them derived from RBC and monocytes; moreover, the RBC-derived EVs were found to be associated with HCT level, reflecting degree of anemia.

In addition, we observed an increase in TF/TFPI ratio together with low EPCR levels in patients’ EVs. Moreover, in splenectomized patients, increased activated-platelet-derived EVs and high EV TF/TFPI ratios were found. This might push the EV hemostatic balance to a procoagulant state, contributing to the well-known prothrombotic tendency in those patients.

We further propose that circulating EV HSP70 is associated with the amount of transfused blood, Hb, erythropoietin and lactate dehydrogenase levels, reticulocyte count and Hb content, indicating ineffective erythropoiesis and hematological disease severity.

Extracellular vesicles concentration, size and composition are potential tools for the diagnosis of various diseases (Revenfeld et al., 2014; Robbins and Morelli, 2014; Camus et al., 2015). However, isolation and characterization methods are not standardized, and a combination of different techniques is recommended. NTA allowed characterization of size distribution and concentration of the entire EV population (50–1,000 nm). The flow cytometry analysis was limited to particles larger than 300 nm, but had the advantage of enabling analysis of multiple fluorescent labels for detection of EV cell origin, and of other membrane antigens, such as coagulation proteins. Applying a combination of methods to determine the concentration, size and cellular origin of EVs in TDT patients, we found differential patterns in Hy and Sp patients. Using NTA, the EV concentration was 3-fold higher in patients than in healthy controls, whereas among patient subgroups, the lowest concentration was observed in the Hy group. The mean size of the EVs indicated that most of them are exosomes that do not significantly differ between patients and healthy controls. However, in Hy patients (**Figure 6**), EV size was significantly smaller than in the other patient subgroups, suggesting a distinct origin and mechanism of EV biogenesis, i.e., intercellular multivesicular bodies vs. cell-surface plasma membrane (Abels and Breakefield, 2016). To the best of our knowledge, this is the first evaluation of EVs in patients with hypersplenism, regardless of the underlying disease, and of EVs by NTA in thalassemic patients. Our results regarding EV concentration, measured by NTA, are in line with previous studies on healthy controls (Gardiner et al., 2013), and on adult patients with sickle cell disease (Marsh et al., 2015).

In addition, in the current study, the concentration of large EVs (MPs), as measured by flow cytometry, was slightly higher in patients than in healthy controls. In line with our results from NTA, the Hy patients had the lowest EV concentrations. Previous studies have demonstrated significantly increased levels of MPs in both NTDT (Pattanapanyasat et al., 2007; Westerman et al., 2008; Chaichompoo et al., 2012) and TDT patients, with higher levels in the splenectomized vs. non-splenectomized patients (Tantawy et al., 2013; Elsayh et al., 2014; Agouti et al., 2015). Only a small fraction, approximately 5% of the large EV population (≥300 nm), can be measured by flow cytometry to identify membrane antigens and external membrane exposure of negatively charged phospholipids, mainly PS. MP formation is usually associated with loss of membrane asymmetry, leading to exposure of negatively charged phospholipids such as PS on the outer leaflet (Thery et al., 2002; Pawlinski et al., 2010). Classically, the identification of MPs has been based on the labeling of vesicles by annexin V, indicating the presence of PS on the outer membrane surface. However, the detection of PS on MPs depends on numerous factors, and not all MPs expose PS on their outer leaflet membrane (Connor et al., 2010; Nielsen et al., 2011; Yuana et al., 2013; Brisson et al., 2017). A recent study reported that an important proportion of EVs, including those larger than 100 nm in size, do not expose PS (Brisson et al., 2017). On the other hand, in the current study, the percentage of annexin-labeled EVs was found to be lower in patients than in controls when we focused on the large EVs, in contrast to previous reports in NTDT patients (Pattanapanyasat et al., 2007; Westerman et al., 2008). The discrepancy between studies is probably related to differences in EV characteristics of patients receiving periodic blood transfusions and those that are non-transfusion dependent. In our study, Hy patients showed the highest percentage of annexin-labeled EVs, as measured by both NTA and flow cytometry (**Figure 6**). This also suggests different mechanisms for EV formation or clearance in TDT patients based on their spleen function, demonstrating the spleen’s important role in the formation/clearance of specific EV populations. This observation is supported by studies in animal models showing that clearance of circulating EVs occurs mainly in the liver, spleen, gastrointestinal tract and lungs, generally due to phagocytosis by macrophages, but it also depends on the EV cell origin (Willekens et al., 2005; Dasgupta et al., 2009; Rautou and Mackman, 2012; Wiklander et al., 2015; Cataldi et al., 2017). Further studies are required to confirm and explain the differences in annexin-labeled EVs between Hy and Sp patients.

In our study, the percentage of RBC-derived EVs was positively correlated to patients’ HCT. RBC in TDT patients are mostly transfused erythrocytes, and RBC MPs originate only in small part from thalassemic erythropoiesis (Agouti et al., 2015). Previous studies in TDT patients have found higher levels of MPs originated from RBC and platelets than in controls (Tantawy et al., 2013; Agouti et al., 2015), most notably in splenectomized patients (Elsayh et al., 2014). The lower amount of RBC EVs found in our study can be explained by the patients’ reduced RBC count, despite receiving regular blood transfusions, and the existence of patients with hypersplenism in the patients’ cohort.

Reduced monocyte-derived EVs were also observed in patients. Monocyte-derived EVs are reported to have pro-inflammatory effects (Wang et al., 2011; Halim et al., 2016). The reduction in monocyte-derived EVs warrants further investigation, as a contribution to the increased tendency for infections can be suggested.

We hypothesize that the lower levels of blood cell-derived EVs found in Hy patients reflect a reduced number of “parent cells of origin” and increased removal of abnormal cells from the circulation—which may affect EV secretion—or increased clearance of these specific EVs by the spleen.

β-TM is considered a chronic hypercoagulable state attributed to several risk factors. Increased levels of platelets, activated platelets, platelet MPs and EV dysregulation also contribute to hypercoagulability in β-TM, particularly after splenectomy (Ruf et al., 1997; Eldor and Rachmilewitz, 2002; Cappellini et al., 2012; Agouti et al., 2015). In the current study, Sp patients showed more than 2-fold higher levels of activated platelet-derived EVs compared to Hy patients and healthy controls, probably contributing to the increased thrombotic risk of patients after splenectomy.

In addition, the evaluation of antigens involved in coagulation on the EV surface reveals specific signatures in β-TM patients. We observed a particularly high TF/TFPI ratio in EVs from Sp patients (2.9-fold that in controls); together with reduced labeling of EPCR EVs (half of the amount in Hy and Sp patients compared to healthy controls). In healthy individuals, EV-TF activity remains undetectable, but in pathological states, TF-bearing EVs may activate the coagulation cascade and induce thrombotic events (Furie and Furie, 2008; Geddings and Mackman, 2013). Increased TF/TFPI ratio can be considered an indicator of hypercoagulable state, and has been found to be associated with increased thrombotic risk, as described in previous studies from our group (Aharon et al., 2009; Tsimerman et al., 2011). The EV TF/TFPI ratio might predict hypercoagulable state in β-TM patients. The percentage of EPCR-labeled EVs was lower in Hy and Sp patients. EPCR, which is predominantly expressed in large-blood-vessel endothelial cells, plays an important role in the protein C pathway in regulating coagulation, inflammation and cytoprotection. Since EPCR EVs are known to preserve functional anticoagulant activity (Perez-Casal et al., 2005; Morel et al., 2009), we postulated that the reduction in the number of EPCR-labeled EVs observed in thalassemic patients can lead to altered hemostatic balance of the activated protein C system, acting as an additional inductive factor of hypercoagulable state in thalassemia patients. Nevertheless, functional coagulation assessments and additional studies are needed to evaluate the consequences these observations and their clinical implications.

Extracellular vesicles content is of great interest due to its role in intercellular communication and exchange of molecular cargo (Kalra et al., 2016; van Niel et al., 2018). The current study demonstrated altered protein content in EVs from TDT patients. Differences in the apoptotic protein profile, and specifically increased HSP70, were found between patients and controls and demonstrated a uniform pattern in the patient subgroups. This is in line with a previous study reporting increased levels of RBC- and platelet-related proteins in MPs from β-TI patients (Chaichompoo et al., 2012), as well as with a study revealing that patient MPs released from β-TI RBC contain hemichromes and HSP70 (Ferru et al., 2014).

HSP70 is important in both normal and β-thalassemia erythropoiesis. In β-thalassemia, HSP70 interacts directly with free α-globin chains and does not translocate to the nucleus; due to the absence of this chaperone’s protection, GATA1 is cleaved and erythroid maturation cannot proceed, aggravating ineffective erythropoiesis (Ribeil et al., 2007; Arlet et al., 2014; Sankaran and Weiss, 2015). HSP70 can be secreted from cells into the circulation via EVs (De Maio and Vazquez, 2013; Yanez-Mo et al., 2015). We therefore considered HSP70 a primary candidate biomarker for the detection of infective erythropoiesis in thalassemic patients. Increased HSP70 levels in EVs were observed in the entire cohort of patients. EV-HSP70 levels were positively correlated with patients’ reticulocyte count and lactate dehydrogenase and erythropoietin levels, and negatively correlated with patients’ Hb and blood-transfusion requirements. Thus HSP70 reflects the degree of ineffective erythropoiesis, hemolysis and anemia severity (**Figure 6**). No correlation was found with iron-overload parameters. EV HSP70 can potentially be used as a novel biomarker for monitoring ineffective erythropoiesis in β-TM, and perhaps in the future, might be considered a therapeutic target (Arlet et al., 2014; Sankaran and Weiss, 2015; Makis et al., 2016).

In summary, β-TM is a chronic hereditary disease characterized by ineffective erythropoiesis and secondary organ dysfunction. EV quantity, size and molecular cargo seem to have a major impact on EVs’ role as intercellular mediators affecting physiological and pathological processes (Camus et al., 2015; Yanez-Mo et al., 2015; Kalra et al., 2016; van Niel et al., 2018).

Extracellular vesicles analyses in thalassemic patients might be used as a biomarker of different disease stages, such as those related to spleen status, hypercoagulability state and ineffective erythropoiesis. This would allow anticipating the risk of possible complications and optimizing personalized treatment.

## Author Contributions

CL was responsible for patient recruitment, treatment and follow-up, designed and performed the experiments, performed the data analyses, and wrote the manuscript. AK was responsible for patient recruitment, treatment and follow-up, and supervised the writing. AR-S assisted in designing the experiments and in analyzing the data. NK performed the electron microscopy analyses and wrote the corresponding Methods section. BB designed and supervised the study, data analysis, and writing of the article. AA designed the study, programmed and assisted with the experiments, evaluated and analyzed the data, and supervised the writing.

## Conflict of Interest Statement

The authors declare that the research was conducted in the absence of any commercial or financial relationships that could be construed as a potential conflict of interest.
